# The Effect of Dissolved Organic Matter (DOM) on the Release and Distribution of Endocrine-Disrupting Chemicals (Edcs) from Sediment under Hydrodynamic Forces, A Case Study of Bisphenol A (BPA) and Nonylphenol (NP)

**DOI:** 10.3390/ijerph16101724

**Published:** 2019-05-16

**Authors:** Jue Ding, Yu Cheng, Zulin Hua, Cong Yuan, Xiaoju Wang

**Affiliations:** 1 Key Laboratory of Integrated Regulation and Resource Development on Shallow Lake of Ministry of Education, College of Environment, Hohai University, Nanjing 210098, China; abaaludj@gmail.com (J.D.); yuancong2018@163.com (C.Y.); wangxiaoju83102659@163.com (X.W.); 2 National Engineering Research Center of Water Resources Efficient Utilization and Engineering Safety, Hohai University, Nanjing 210098, China; 3 Center for Hydrosciences Research, School of Earth Sciences and Engineering, Nanjing University, Nanjing 210098, China; nju_chengyu@126.com

**Keywords:** endocrine-disrupting chemicals, hydrodynamic intensity, dissolved organic matter, colloid-bound phase, soluble phase

## Abstract

Endocrine-disrupting chemicals (EDCs) that exist in the aquatic system bring severe environmental risks. In this study, we investigate the dissolved organic matter (DOM) effect on the release and distribution of EDCs under varied hydrodynamic conditions. A water chamber mesocosm was designed to simulate the hydrodynamic forces in a shallow lake. The contents of bisphenol A (BPA) and nonylphenol (NP) in colloid-bound and soluble phases were measured under four increasing hydrodynamic intensities that were 5%, 20%, 50%, and 80% of the critical shear stress. The total BPA and NP contents in overlying water grew linearly with the hydrodynamic intensity (*R*^2^ = 0.997 and 0.987), from 108.28 to 415.92 ng/L of BPA and 87.73 to 255.52 ng/L of NP. The exponential relationships of EDC content and hydrodynamic intensity in soluble phase (*R*^2^ = 0.985 of BPA and 0.987 of NP) and colloid phase (*R*^2^ = 0.992 of BPA and 0.995 of NP) were also detected. The DOM concentrations in colloid-bound phase (cDOM) and in soluble phase (sDOM) were measured and the linear relationships with BPA content (*R*^2^ = 0.967 of cDOM and 0.989 of sDOM) and NP content (*R*^2^ = 0.978 of cDOM and 0.965 of sDOM) were detected. We analyzed the ratio (αDOM) of sDOM and cDOM that grew logarithmically with the hydrodynamic intensity (*R*^2^ = 0.999). Moreover, the ratio (αEDCs) of BPA and NP contents in soluble and colloid-bound phases varied differently with αDOM. The results suggested that BPA tended to be in the soluble phase and NP tended to be in the colloid-bound phase due to the increasing value of αDOM.

## 1. Introduction

Endocrine-disrupting chemicals (EDCs) are extensively used in plastics, detergent, and electronic equipment, resulting in wide detection of EDC residues in the environment [1]. As EDCs can disrupt the human endocrine system, large discharges of anthropogenic EDCs (such as bisphenol A (BPA) and nonylphenol (NP) into natural water bodies through municipal wastewater emission bring severe environmental risks [2]. According to previous studies, EDCs have been found in aquatic systems in various phases, including dissolving in water, adsorbing on suspended particulate matter or sediment, and accumulating in aquatic organisms [1,3]. The transportation mechanisms of EDCs though multi-media of aquatic systems are significantly important for understanding their environmental fates [4].

BPA and NP have high lipophilicities (BPA with log K_ow_ = 3.18 and NP with log K_ow_ = 5.76), and tend to sorption to the organic matter (OM), which is the essential part of sediment [1,5]. The sorption of EDCs and OM involves hydrogen bonding, ligand exchange, and hydrophobic interaction, etc. The dissolved organic matter (DOM) acts as the main medium for the transport of BPA and NP in aquatic systems [5], since DOM displays high capacity of BPA and NP binding with its natural ligands and adsorption sites. Under certain disturbances, DOM can diffuse from sediment porewater into water columns, or can be adsorbed on the sediment particles, causing a redistribution of EDCs in aquatic systems [4,6]. Subsequently, the migration range and rate of BPA and NP in aquatic systems is extended by binding with DOM.

The distribution and transportation of EDCs in aquatic systems is a highly complex process involving adsorption, desorption, dissolution, diffusion, and degradation. Although hydrodynamic forces are ubiquitous in natural water bodies [7], few studies have focused on the influence of hydrodynamic forces on the distribution and transportation of EDCs. Especially in shallow lakes, hydrodynamic forces can facilitate porewater advection and particle resuspension, causing physiochemical changes in water columns and sediments [8,9]. The shear stress generated from hydrodynamic forces can disrupt the diffusion boundary layer in sediment–water interfaces (SWIs), and alter the porewater pressure in sediments, which would cause the interchange between porewater and water columns [9,10,11]. In previous studies, resuspension of particulate matter from sediment under hydrodynamic forces has been regarded as a main reason for pollution release into water columns [7]. However, DOM is considered to be a more efficient sorbent for EDCs, in comparison to suspended particles and sediments [12,13]. With high water solubility, DOM binding with EDCs can efficiently migrate though porewater from sediments into water columns under hydrodynamic forces [5]. Moreover, EDCs in sediments can also be affected by the phases that contain DOM [3]. In natural aquatic systems, the dissolved part of a substance can be further subdivided into colloids and soluble phases. Colloids are the particles within the size range of 0.01 to 1 μm [1]. The DOM in colloid-bound phase (cDOM) can influence the distribution of EDCs differently than the DOM in soluble phase (sDOM) [1,14].

In this paper, we aim to investigate the effect of DOM on the phase distribution and transportation of BPA and NP under different hydrodynamic forces in an aquatic system. We designed a series of laboratory simulation experiments using hydrodynamic water chamber mesocosms. The objectives of this study were to (1) construct a water chamber mesocosm to simulate the hydrodynamic forces in a shallow lake area; (2) study the distribution of the EDCs in the aquatic system; (3) investigate the transportation mechanism of EDCs with the assistance of DOM.

## 2. Materials and Methods

### 2.1. Sample Collection and Characterization

Sediment samples were collected from the sediment in Chaohu Lake, Anhui province, China. The background concentrations of BPA and NP in the sediment of Chaohu Lake were relatively high, with the initial concentrations of 114.56 ng/g for BPA and 105.73 ng/g for NP. After the collection, the sediment samples were stored in coolers and immediately transported to a laboratory in Hohai University, and were stored in the refrigerator under 4 °C. The sediment samples were sieved using a 0.6-mm pore-size mesh.

### 2.2. Experimental Setup

**Figure 1:**
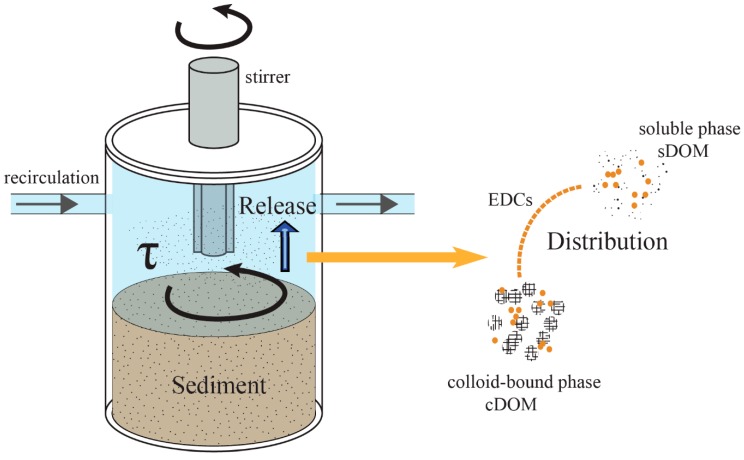
The scheme of the hydrodynamic water chamber mesocosm. EDCs: endocrine-disrupting chemicals; DOM: dissolved organic matter; sDOM: DOM in in soluble phase; cDOM: DOM in colloid-bound phase.

A hydrodynamic water chamber mesocosm was designed to simulate the hydrodynamic forces in a shallow lake (Figure 1). The mesocosm is a cylinder type chamber with a set of stirrers on the top. All parts of the chamber were made of polycarbonate to minimize the adsorption effect from the setup. The driving device consisted of a spinning disk and an inverter motor. The inverter motor controlled the rotation rate of the spinning disk, providing adjustable shear stresses over the SWI [9]. The sediment in the bottom of the chamber was with 10 cm deep. The chamber was connected to a water reservoir through a peristaltic pump. The peristaltic pump exchanged the water between the chamber and reservoir to keep homogenous distribution of dissolved and particulate matter in the overlaying water during the experiments. The total volume of the overlaying water was 5 L.

### 2.3. Hydrodynamic Conditions

**Figure 2:**
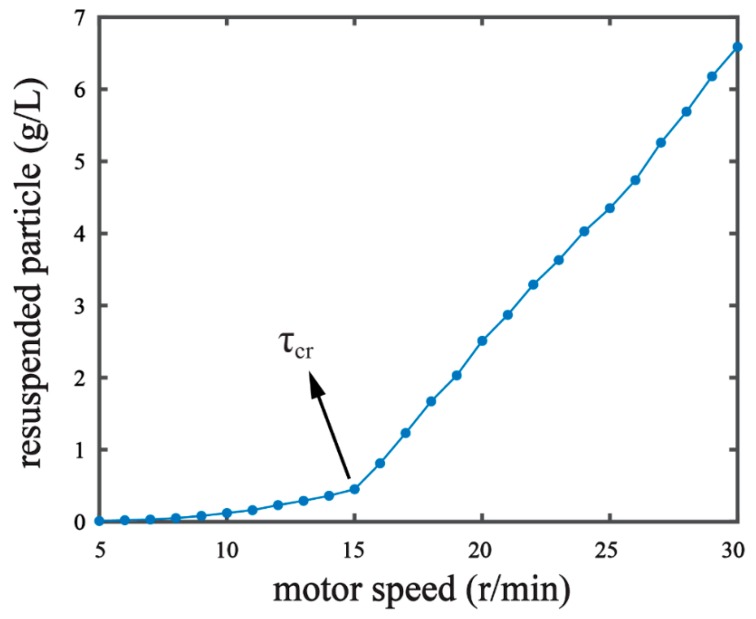
Identifying critical shear stress (*τ_cr_*) of the sediment by measuring resuspended particles in response to stepwise increases of the motor speed of the experiment setup. *τ* = *τ_cr_* was recognized when the motor speed reached 15 r/min.

Four hydrodynamic conditions were chosen for the experiments based on the shear stress of hydrodynamic forces. When the shear stress is higher than the critical shear stress (τ_cr_), sediment particles will resuspend in a water column and the EDCs in particle-bound phase will be brought into the water column. Since our study focused on the DOM effect, the effect of EDCs in particle-bound phase should be eliminated [1,15]. Thus, the hydrodynamic force in our experiments cannot result in an obvious resuspension effect in the overlying water, the shear stress of which should be below τ_cr_ of the sediment [16]. The τ_cr_ generated by the setup is calculated based on the following equations [10,17]:

(1) $$\tau_{cr}=\theta_{cr}g\left(\rho_{s}-\rho \right)d$$

(2) $$\theta_{cr}=\frac{0.30}{1+1.2D_{*}}+0.55\left[1-\exp\left(-0.020\right)D_{*}\right]$$

(3) $$D_{*}={\left[\frac{g\left(\rho_{s}/\rho -1\right)}{v^{2}}\right]}^{1/3}d$$

where *θ_cr_* is the threshold Shields parameter; g is the gravitational acceleration due to gravity; *ρ_s_* is the mass density of sediment (kg/m^3^); *ρ* is the mass density of water (kg/m^3^); D_*_ is the dimensionless grain size; *d* is the grain diameter (m); and υ is the kinematic viscosity of the water (m^2^/s). In our pre-experiments, we observed the resuspension phenomenon by measuring the resuspended particle content under a series of different hydrodynamic forces [16,18]. The resuspended particle content increased with the motor speed of our experiment setup (Figure 2). When the motor speed reached over 15 r/min, a significant increase in particle content was observed, suggesting the critical condition of $\tau =\tau_{cr}$. The resuspended particle in the water collected was measured on a laser particle analyzer (Mastersized 2000, Malvern Panalytical Ltd., Malvern, UK) [7] in order to determine the median diameter (*d_50_*) of the resuspended particle. The *d_50_* of the resuspended particle under the critical condition was 0.016 mm. With the d_50_ value, *τ_cr_* = 0.0228 Pa was derived by the Equations (1)–(3). We set the shear stress in our experiments as $\tau =\theta \tau_{cr}$, where *θ* is the percentage of the critical shear stress. The shear stress conditions of 5%, 20%, 50%, and 80% of the critical shear stress were chosen for experiment G1, G2, G3, and G4, respectively (Table 1).

### 2.4. Experimental Procedure and Sampling

The homogenized sediment was filled into the chamber for a 10-cm-deep sediment bed and 5.0 L volume of deionized water was put into the water reservoir. The peristaltic pump slowly extracted the water from the reservoir into the chamber until the water level of the chamber reached 20 cm. After the driving devices were set according to the right parameters, the experiments were initiated. The four experiments were continuously run for 24 h, and the systems of the four experiments reached steady state by then [7,18]. The water sample and sediment sample collections were taken when the experiments reached steady state. Redox potential (pE), pH, dissolved oxygen (DO), and temperature of the water were measured directly in the setups with a probe (HQ30d, Hach, Danaher, Washington, D.C., USA).

### 2.5. Sample Analysis

#### 2.5.1. Sample Fractionation

The water samples were adjusted to pH 3 with 1 mol/L hydrochloric acid and filtered through 0.45-μm quartz fiber filters using a vacuum filtration [4,8]. The filtrate was divided into two components that were colloid-bound and dissolved phases using an ultrafiltration system (Millipore Pellicon 2, Merck Millipore, Darmstadt, Germany) [1]. The colloid-bound phase was the retentate flow separated by the filtration system, while the dissolved phase was the permeate flow [4].

#### 2.5.2. Extraction of EDCs from Water Samples

Solid phase extraction and derivation were used for the water sample (colloid-bound and soluble phases) extraction. The water sample was passed through an HLB cartridge (500 mg, 6 cc, Waters, Milford, MA, USA) that was pre-conditioned three times with 5 mL of MTBE, 5 mL methanol and finally 5 mL of ultrapure water in sequence [1]. Additionally, the cartridge was washed with 10 mL of 10% methanol solution to remove interferences. Afterwards, the target EDCs were eluted from the cartridge with 6 mL of methanol and 6 mL of dichloromethane [1,19]. The extract was dehydrated with anhydrous sodium sulfate and concentrated to 1 mL in a rotary evaporator. The refined extract was derived using bis (trimethylsilyl) trifluoroacetamide (BSTFA 1% TMCS) and heated in an air oven at 70 °C for 30 min [20]. Then, the solution of extract was evaporated under a nitrogen gas stream. Finally, the derived extract was reconstituted to 1 mL volume with ethyl acetate.

#### 2.5.3. EDCs Extraction of Sediment Sample

The sediment samples were dried in the lyophilizer and then passed through a 100-mesh sieve [8]. Acetone-ethyl acetate was prepared as the extraction solvent for the solid sample. A total of 0.5 g (dry weight) of the solid sample was mixed with 5 mL of solvent, ultrasonicated at 42 kHz for 15 min, centrifuged at 6000 rpm for 15 min, and the supernatant was decanted [21]. Next, the solid sample was extracted successively two times with the solvent of acetone and then successively two times with the solvent of ethyl acetate [21]. The supernatants were combined and evaporated under the nitrogen gas stream with a water bath at 40 °C until reaching a volume of about 1 mL. The concentrated extract was re-dissolved into 500 mL of de-ionized water and the pH was adjusted to 3 [21,22]. The rest of the extraction and derivatization procedures were the same as those of water sample in Section 2.5.1.

#### 2.5.4. GC-MS Analysis

EDCs were analyzed using a GC-MS system (Thermo DSQ II, ThermoFisher, Waltham, MA, USA) operating in the electron capture negative ionization mode. The column was an HP-5MS capillary column (30 m × 0.25 mm, 0.25 mm film thickness). The carrier gas was high-purity helium at a flow rate of 1 mL/min. One microliter of the derivatized sample was injected into the gas chromatograph in splitless mode [1]. The injection port and transfer line were held at 280 °C. The ion source temperature was 300 °C. The GC oven temperature program started at 100 °C for 1 min, then increased by 10 °C/min to 200 °C. Afterwards, the oven temperature increased by 5 °C /min to 280 °C, and then was maintained for 2 min. The limits of detection (LOD) of BPA and NP were respectively 0.01 and 0.1 ng/L. The limits of quantification (LOQ) of BPA and NP were respectively 0.2 and 0.3 ng/L. The recoveries of BPA and NP were respectively 95.1% and 93.6%.

### 2.6. Statistical Analysis

In this paper, *t*-test and nonparametric Kruskal–Wallis test methods were performed on the data with significant differences indicated by *p* < 0.05. We used Python 3.7 SciPy 1.2.0 (www.python.org to process the statistical approaches and Inkscape 0.92.4 (www.inkscape.org) to process the graphs.

## 3. Results and Discussion

### 3.1. Physiochemical Properties of the Water Column

The physiochemical properties including DO, pH, pE, and temperature were measured in experiment G1, G2, G3, and G4, the data of which is shown in Table 2. As the hydrodynamic intensity increased from G1 to G4, the DO concentration in water increased from 8.2 mg/L in G1 to 9.3 mg/L in G4. Higher hydrodynamic intensity brought more oxygen dissolving in overlying water. The pH value decreased from 8.44 in G1 to 8.28 in G4, while the pE value decreased from 174 mV in G1 to 169 mV in G4. The anaerobic degradation process within the sediment may generate acid and reducing material. The increased intensity of hydrodynamic disturbance can bring more acid and reducing material from sediment into overlying water, causing a decrease in pH and pE [9].

### 3.2. The Release EDCs in an Aquatic System under Hydrodynamic Forces

#### 3.2.1. Distributions of EDCs in Overlying Water

**Figure 3:**
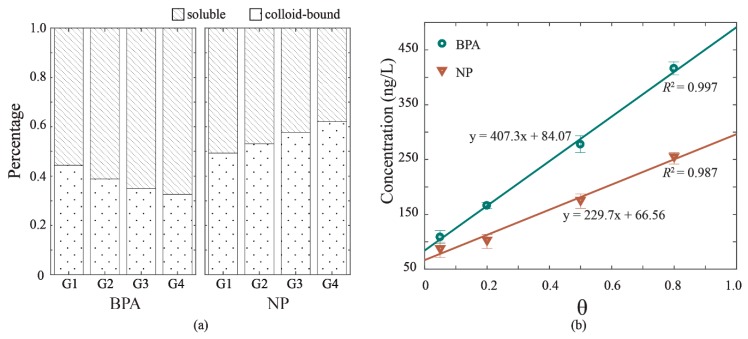
The release of bisphenol A (BPA) and nonylphenol (NP) in overlying water under hydrodynamic forces. (**a**) Distributions of BPA and NP in two phases: soluble and colloid-bound. (**b**) The correlations of total concentrations in overlying water and hydrodynamic intensity (θ).

The release of BPA and NP to overlying water of experiment G1, G2, G3, and G4 varied with the hydrodynamic intensity, with respective mean total concentrations of 108.28, 165.82, 277.48, and 415.95 ng/L of BPA, and 87.73, 103.15, 175.91, and 255.52 ng/L of NP. These values are within the ranges of those detected in natural water bodies [23,24,25]. The linear relationships to hydrodynamic velocity were found for both BPA concentration and NP concentration, with *R*^2^ = 0.997 and *R*^2^ = 0.987 (Figure 3b). The result suggested that higher hydrodynamic intensity caused larger releases of BPA and NP to overlying water. The concentrations of BPA and NP were detected in two phases of overlying water as soluble phase and colloid-bound phase, the percentages of which are depicted in Figure 3a. The soluble phase contained the highest proportion of BPA in two phases, which increased from 0.57 in G1 to 0.67 in G4. Accordingly, the colloid-bound phase of BPA decreased from 0.44 in G1 to 0.33 in G4. The distribution of NP under hydrodynamic forces behaved differently from that of BPA. The colloid-bound phase of NP increased from 0.49 in G1 to 0.62 in G4, and the soluble phase of NP decreased from 0.51 in G1 to 0.38 in G4. The organic-carbon-normalized sorption coefficient (log K_oc_) values of BPA and NP were 4.42 and 4.98, respectively, suggesting a stronger sorption effect of NP and DOM than that of BPA and DOM. Also, the log K_ow_ values of BPA and NP are diverse (3.18 for BPA and 5.76 for NP) [1,26]. According to previous studies [26,27,28,29,30], sorption mechanisms play a more important role than hydrophobic interaction in the sorption EDCs sorption (i.e., hydrogen bonding and π-π interactions). The results confirmed that NP can be more adsorbed to colloids with high capacities and concentrations of organic matter [26].

#### 3.2.2. The Correlation of Hydrodynamic Intensity and EDCs Content

**Figure 4:**
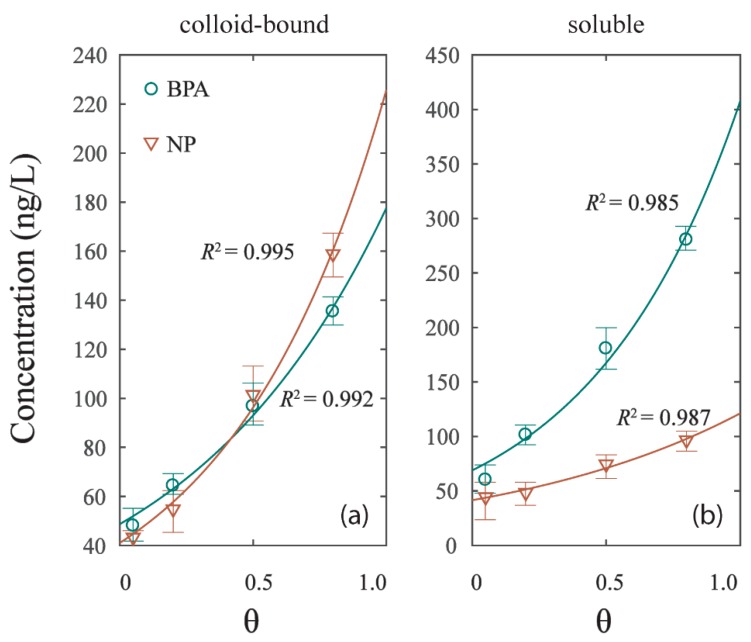
The correlation of EDCs concentration in different phases and hydrodynamic intensity (θ). (**a**) Colloid-bound phase. (**b**) Soluble phase.

The contents of BPA and NP of two phases (colloid-bound and soluble) were found to have a significant correlation (*p* < 0.05) with the hydrodynamic intensities of experiment G1, G2, G3, and G4 (Figure 4). The relationships can be deduced as follows:

(4) $${\left[C\right]}_{phase}=k_{1}\times \exp\left(k_{2}\times \left[\theta \right]\right)$$

where *k*_1_ and *k*_2_ are coefficients that were calibrated using the nonlinear fitting technique in Table 3, and [C]_*phase*_ represents the concentrations of BPA or NP in colloid-bound and soluble phases; [*θ*] represents the hydrodynamic intensity of the experiment with the shear stress $\tau =\theta \tau_{cr}$. All data were well fitted, with *R*^2^ > 0.980. The concentration of colloid-bound and soluble phases in the four experiments were respectively 40–160 ng/L and 40–280 ng/L, significantly growing with the hydrodynamic intensity. The result implied that the colloid-bound and soluble phases were sensitive to the hydrodynamic forces [31]. While the mechanism of particles liberation is a resuspension effect, the main effect of EDCs release under hydrodynamic forces is the DOM inducement to colloid-bound and soluble phases [26]. The release curve of BPA in soluble phase is steeper than that of NP, while the release curve of NP in colloid-bound phase is steeper than that of BPA (Figure 4a,b). The result suggested that the dissolve effect of BPA grew faster with the rising hydrodynamic intensity than that of NP. As per the colloid-bound phase, the NP content under low hydrodynamic intensity was lower than the BPA, and subsequently overpassed BPA under high hydrodynamic intensity. This phenomenon may be caused by the changes of DOM distribution in colloid-bound and soluble phases under hydrodynamic forces.

#### 3.2.3. Mass Balance of the Influx and Efflux of EDCs within the Aquatic System

The mass balance between the overlying water and sediment can be taken as a sign of the stability of our experiments. The mass efflux from sediment (SEF) can be estimated from the BPA and NP concentrations of sediment in the initial and final experiment stage. The mass influx into overlying water (WIF) can be estimated from the increase of BPA and NP concentrations in overlying water [1,7]. Then, the mass balance can be measured as the ratio (WIF/SEF) of WIF and SEF (Table 4). The WIF/SEF of BPA was around 0.97 to 0.99. Meanwhile, the WIF/SEF of NP was around 0.90 to 1.05. In all four experiments, the mass of BPA and NP transported between water and sediment was greater than that of NP. Although the mass transport between the sediment and overlying water increased with the hydrodynamic intensity, the mass balance was stable in all four experiments.

### 3.3. The Effect of DOM for EDCs Release under Hydrodynamic Force

The EDC contents increased with the organic contents of colloid-bound and soluble phases. The mean average concentration of organic matter (TOC) in the colloid-bound phase was steadily around 0.77 mg/g. Thus, the content of colloid in overlying water can be quantified as the organic matter content of colloid dissolving in water (cDOM).

**Figure 5:**
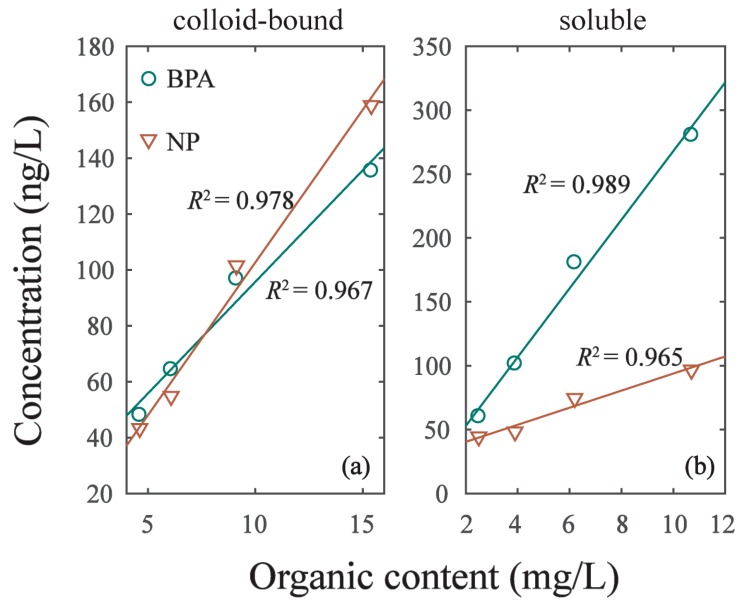
The correlation of EDCs concentration in two phases and its organic content. (**a**) Colloid-bound phase. (**b**) Soluble phase.

As the intensity of hydrodynamic disturbance increased from experiment G1 to G4, the cDOM in overlying water increased from 4.62 mg/L to 15.4 mg/L. The linear correlation (y = k_3_ × x + k_4_) of cDOM and EDCs was *R*^2^ = 0.967 in BPA and *R*^2^ = 0.978 in NP (Figure 5; Table 5). Meanwhile, the DOM in soluble phase (sDOM) increased with the intensity of hydrodynamic disturbance as well. Additionally, we found the linear correlation (y = k_3_ × x + k_4_) of sDOM and EDCs was *R*^2^ = 0.989 in BPA and *R*^2^ = 0.965 in NP. The linear slope (k_1_) of BPA and NP in Table 5 varied with phases. The K_1_ of BPA in colloid-bound phase was 7.99 and increased to 26.89 in soluble phase. The K_1_ of NP in colloid-bound phase was 10.93 and decreased to 6.68 in soluble phase. This result implied that BPA can be more easily adsorbed on sDOM of soluble phase, while NP can be more easily adsorbed on cDOM of colloid-bound phase. The sorption of BPA and NP on DOM was significantly different due to their different characteristics, resulting in their diverse redistribution in colloid-bound and soluble phases [1,32].

**Figure 6:**
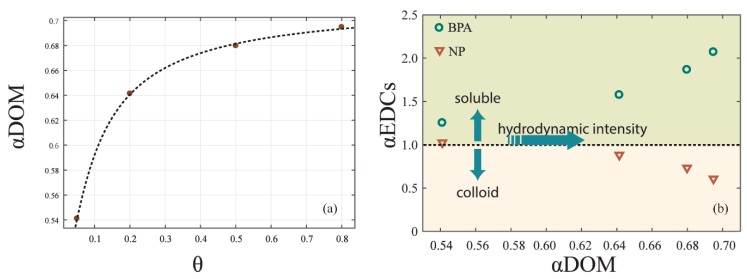
The effect of DOM on the EDCs distribution. (**a**) The correlation of αDOM and hydrodynamic intensity. (**b**) The correlation of αEDCs and α_DOM_ under hydrodynamic forces.

In order to quantify the different effects of cDOM and sDOM in the redistribution of EDCs, we analyzed the relationship between the ratio (αDOM) of sDOM and cDOM and hydrodynamic intensity. A logarithmic relationship (Figure 6a) can be depicted as follows:

(5) $$\left[\alpha_{DOM}\right]=k_{5}\times \;\exp\left(-k_{6}\;\times \;\left[\theta \right]\right)+k_{7}$$

where *k*_5_, *k*_6_, and *k*_7_ are coefficients that were calibrated as −0.212, 0.086, and 0.504, respectively; [θ] represents the hydrodynamic intensity of the experiment with the shear stress $\tau =\theta \tau_{cr}$. The equation was well fitted, with *R*^2^ = 0.999.

As the αDOM was below 1.00, there was more cDOM in the overlying water than sDOM. Under hydrodynamic forces, the increase of sDOM was higher than that of cDOM, leading a growth of αDOM. However, since the shear stress caused by hydrodynamic forces was always under the critical shear stress for the sediment resuspension, αDOM converged to 0.70, which was still below 1.00. As a result, sDOM formed no more than 41.2% of DOM in overlying water under the resuspension critical shear stress.

Furthermore, we analyzed the ratio (αEDCs) of EDCs contents in soluble and colloid-bound phases. With αEDCs > 1.0, more EDCs content was in the soluble phase than in the colloid-bound phase, and vice versa. αEDCs of BPA and NP changed with αDOM in different ways (Figure 6b). As αDOM increased from 0.54 to 0.69, which was near the upper limit of 0.70, αEDCs of BPA increased from 1.25 to 2.07, while αEDCs of NP decreased from 1.03 to 0.61. This result emphasized that the proportion of DOM in soluble and colloid-bound phase affects the redistribution of EDCs in overlying water [33]. With αDOM increasing with the hydrodynamic intensity, BPA tended to be in the soluble phase and NP tended to be in the colloid-bound phase.

## 4. Conclusions

In this paper, we performed four groups of experiments to investigate the DOM effect on the release and distribution of EDCs under hydrodynamic forces. With the mesocosm providing variations in the hydrodynamic intensity, the BPA and NP contents in colloid-bound and soluble phase of overlying water were measured. Under the increasing intensity of hydrodynamic forces, the total amount of BPA and NP released from sediment was significantly enhanced. However, the distribution patterns of BPA and NP were significantly different. With the diversity of K_oc_ and K_ow_ values, the percentage of BPA content in soluble phase increased and NP content in soluble phase decreased. NP with higher value of K_oc_ tended to be adsorbed on colloids, and this behavior was enhanced by hydrodynamic forces. The logarithmic correlations of hydrodynamic intensity and EDCs contents were discovered in two phases. Moreover, the ratio of DOM in soluble phase and colloid-bound phase (αDOM) grew with the hydrodynamic intensity, implying that sDOM rises more quickly than cDOM. This result explained the increasing αEDCs of BPA and decreasing αEDCs of NP under hydrodynamic forces. As the shear stress in the experiments was below the critical shear stress, the upper limit value for αDOM was found and the variation range of αEDCs was limited. This study suggests that the DOM effect on the release and distribution of EDCs with various characteristics in aquatic systems should be further studied.

## Figures and Tables

**Table 1:** Hydrodynamic conditions chosen for the experiments.

Series	*θ* *	Shear Stress (Pa)
**G1**	0.05	0.0011
**G2**	0.20	0.0046
**G3**	0.50	0.0114
**G4**	0.80	0.0182

* *θ* is the percentage of the critical shear stress.

**Table 2:** The physiochemical properties of the water column in the four experiments.

Series	DO * (mg/L)	pH	pE (mV)	Temperature (°C)
**G1**	8.2	8.44	174	24.3
**G2**	8.5	8.36	173	24.5
**G3**	8.8	8.33	171	24.4
**G4**	9.3	8.28	169	24.3

* DO is dissolved oxygen.

**Table 3:** Coefficients of nonlinear fitting of hydrodynamic intensity and EDCs content.

Phases	EDCs	k_1_	k_2_	*R* ^2^
colloid-bound	BPA	48.73	1.29	0.992
	NP	41.06	1.70	0.995
soluble	BPA	68.89	1.78	0.985
	NP	41.52	1.07	0.987

**Table 4:** The mass balance between sediment efflux and water influx.

Series	BPA			NP		
	SEF * (ng)	WIF ** (ng)	WIF/SEF ***	SEF (ng)	SEF (ng)	WIF/SEF
**G1**	550	541	0.98	418	439	1.05
**G2**	836	829	0.99	572	516	0.90
**G3**	1408	1387	0.99	946	880	0.93
**G4**	2134	2080	0.97	1364	1278	0.94

* SEF represents the mass efflux from sediment. ** WIF represents the mass influx into overlying water. *** WIF/SEF represents the ratio of WIF and SEF.

**Table 5:** Coefficients of nonlinear fitting of DOM and EDCs content in two phases.

Phase	EDCs	k_3_	k_4_	*R* ^2^
colloid-bound	BPA	7.985	15.86	0.978
	NP	10.930	−6.656	0.988
soluble	BPA	26.89	−0.902	0.989
	NP	6.678	27.07	0.965
